# Vesicoureteral Reflux Diagnosis After Hospitalization for Acute Cystitis and Pyelonephritis

**DOI:** 10.7759/cureus.38216

**Published:** 2023-04-27

**Authors:** Shay Reinke, Zairha Snider

**Affiliations:** 1 Medicine, Edward Via College of Osteopathic Medicine, Blacksburg, USA; 2 Pediatrics, Edward Via College of Osteopathic Medicine, Blacksburg, USA

**Keywords:** urosepsis, voiding cystourethrogram, recurrent urinary tract infection, pediatric urinary tract infection, pyelonephritis, vesicoureteral reflux

## Abstract

Children with recurrent fevers in a short period of time need to be worked up to identify the underlying cause. Fevers in children and infants can be from many different sources. Vesicoureteral reflux (VUR) is an anatomical and physiological anomaly in children that can lead to retrograde urine flow from the bladder back into the distal ureters. This retrograde flow can cause distention, scarring, and recurrent infections including urinary tract infections (UTIs) and pyelonephritis. Identification of multiple UTIs in a short period of time should raise suspicion for a more complex pathology such as VUR and requires a more thorough workup. This workup is needed for both diagnosis and treatment. The patient in this report was seen by physicians in the emergency department, pediatric intensive care unit, nephrology, and her pediatrician. If surgery is needed, a urologist would also be involved. This report will discuss the pathophysiology of VUR and associated pathologies, diagnostic approach, medical and surgical treatment modalities, as well as prognosis.

## Introduction

Vesicoureteral reflux (VUR) can be caused by either abnormal positioning of the ureter into the bladder or by poor integrity of the ureterovesical junction. Multiple studies have shown this by identifying specific genes that are involved with the formation and growth of the ureter, attachment of the ureter to the bladder, and development of the kidney [1,2]. These anatomic variances can lead to reflux of urine which increases the risk of infections of the entire urinary tract and possible urosepsis if left untreated [3]. Treatment of VUR often includes antibiotics to eliminate a current infection and work prophylactically against future infections. However, surgical intervention may be needed for recurrent urinary tract infections (UTIs), persistent urine reflux, or substantial scarring in the kidneys or ureters [1,4].

The improper positioning or decreased integrity of the ureterovesical junction can be broken down into five grades with increased severity [1]. The international grading system for VUR: Grade I is reflux only into the non-dilated ureter, grade II is reflux into the ureter and the renal pelvis without dilatation, grade III is reflux with mildly dilated ureter and calyces, grade IV is reflux with the tortuous and moderately dilated ureter with blunting of renal fornixes while the papillary impression is preserved, and grade V is reflux with the tortuous and severely dilated ureter, dilatation of calyces with loss of fornixes, and papillary impression. VUR grade can help guide treatment as lower grades (I-III) are more likely to spontaneously resolve with medication prophylaxis while higher grades (IV-V) may require surgical management [1,5].

VUR is diagnosed using a voiding cystourethrogram (VCUG). The bladder is filled with contrast which helps with visualization under fluoroscopy. The American Academy of Pediatrics guidelines indicate a VCUG if they have a febrile UTI with a renal ultrasound consistent with hydronephrosis or recurrent UTI [6].

VUR is present in 30%-40% of pediatric cases of UTI and 1%-2% of the entire population of children. VUR can be divided into primary and secondary VUR. Primary VUR is more common and is described as a congenital condition that occurs with abnormal development, and secondary VUR is acquired due to increased pressure in the bladder due to an outflow obstruction [7]. In screening studies, an increased prevalence in siblings and offspring led to investigation [8]. Studies have shown several genes that are linked with ureteric bud branching, elongation, and insertion into the bladder to have a part in the development of VUR. Embryologically, these genes are for transcription factors that are produced by the pronephros and mesonephros and are ultimately responsible for the formation of the metanephrogenic mesenchyme and metanephros development. The specific genes are Lim-1, Pax-2, Eya-1, and Foxc-1. After the mesenchyme is formed, it will give off a neurotropic factor that will cause the Wolffian duct to give off a uretic bud [2]. When this embryological pathway is altered, the formation of anatomic variances can lead to the retrograde reflux of urine.

Children usually are not diagnosed with VUR until they present with recurring UTI or have an examination for the cause of hydronephrosis. Higher-grade VUR that leads to reflux increases the likelihood of a UTI. VUR tends to resolve spontaneously and so can usually be treated medically without the need for surgical intervention [1,9]. Antibiotic prophylaxis to prevent UTI is recommended for all patients with VUR regardless of grade. The commonly used prophylactic antibiotic regimens include amoxicillin, amoxicillin-clavulanic acid, cefadroxil, cephalexin, nitrofurantoin, or trimethoprim-sulfamethoxazole [10]. If the symptoms continue after medical treatment and prophylaxis, surgery may be necessary. The surgery can be done both open and endoscopically with the open procedure having a higher success rate of 98.1% compared to 83.0% [1].

## Case presentation

The patient is a female infant that was first seen at two days old for her newborn exam and was followed until her most recent sick visit at 20 months of age. Her newborn visit was unremarkable at two days old. She had normal urine output and normal external genitalia on the physical exam. Her one-month well-child check was also unremarkable. At approximately two months of age, the patient’s mother took her to the emergency department due to an at-home temperature of 102°F. In the emergency department, she had a temperature of 101.9°F and was tachycardic at 170 bpm with all other vitals being within normal limits for her age. Chest x-rays, Influenza A and B, COVID-19, and RSV were all negative. UA showed signs of infection and mild hematuria. The UA showed over 100,000 colonies/mL of E. coli. The patient was discharged home with a diagnosis of acute cystitis with hematuria and a prescription of amoxicillin.

**Figure 1:**
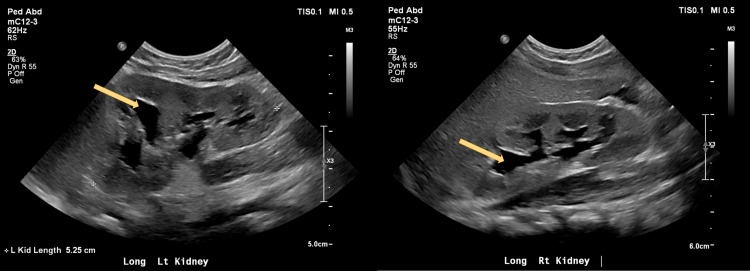
Renal ultrasound showing hydronephrosis in bilateral kidneys

**Figure 2:**
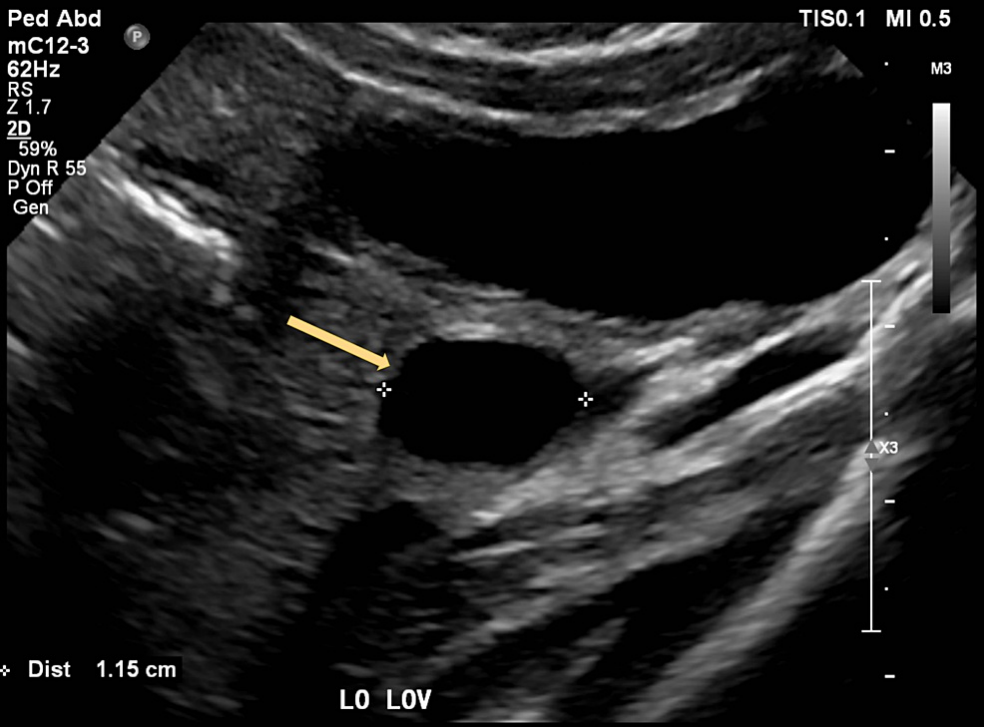
Ultrasound of left ovarian cyst

The next morning the patient presented to her PCP with complaints of emesis and runny stools after the administration of amoxicillin. The PCP repeated a UA catheter, and the UTI was still present. The antibiotic was discontinued and replaced with cephalexin. Due to concerns about bacteremia, the child was admitted to the hospital that same day for further evaluation. While admitted, she received IM ceftriaxone and had no fever recurrence. Renal ultrasound showed mild bilateral hydronephrosis as well as an incidental left ovarian cyst as seen in Figures 1, 2. She was discharged home the next day with continued cephalexin.

**Figure 3:**
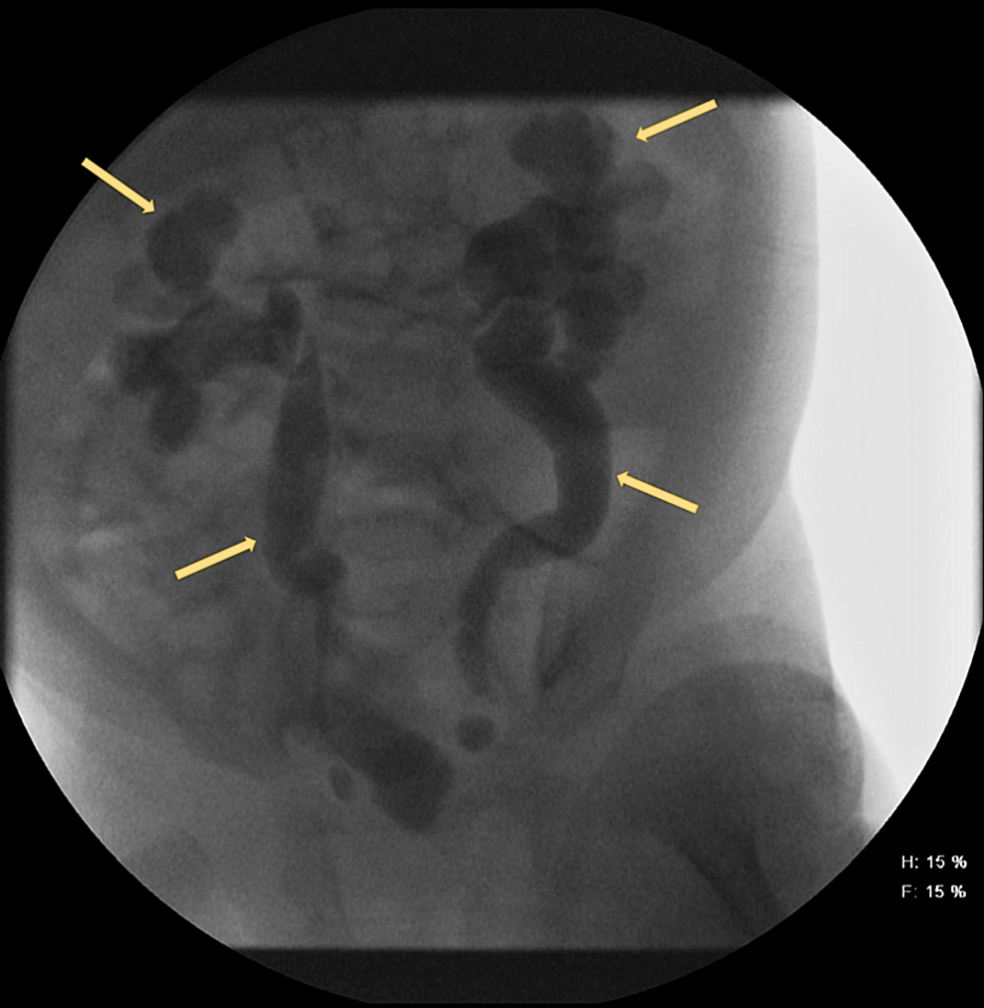
VCUG reveals dilated ureters and dilated renal calyces distorting the normal renal anatomy. VCUG - voiding cystourethrogram

She followed up with her PCP later that week with no complaints. Fever was controlled and the mother was informed to return in two weeks for a repeat ultrasound or to return sooner if any problems arose. Less than two weeks later, the mother brought the girl to the emergency department with another UTI. The patient was admitted. During this admission, she had repeat ultrasounds and a VCUG, shown in Figure 3, that demonstrated a grade V VUR.

After discussion with nephrology, the patient had an appointment scheduled and was placed on prophylactic Bactrim (trimethoprim-sulfamethoxazole). Five months later, she was seen by nephrology. She had recently had another febrile UTI and the Bactrim was switched to Augmentin (amoxicillin-clavulanic acid). It was decided that with the good response to antibiotics, surgery can be withheld, and treatment medically will suffice for the present time. The patient is to follow up with pediatric nephrology in 6 months and continue regular visits with her pediatrician.

## Discussion

This case demonstrates a case of VUR after multiple UTIs. Patients with VUR are more susceptible to UTIs due to the retrograde flow of urine and the bladder not being able to expel the bacteria. The symptoms that should alert the parents that their child may need to be seen by a provider would include fever, emesis, and change in bowel and urinary frequency. A fever can have many underlying causes which is why it is important to rule out ear infections and respiratory infections. It is also necessary to evaluate the urine. This patient was diagnosed with acute cystitis in the emergency department and was discharged home with an antibiotic. However, she did not tolerate the antibiotic and the infection persisted. After hospitalization and further evaluation, it was determined that she had hydronephrosis. After another UTI, a VCUG revealed grade IV/V VUR.

A case as complex as this shows the importance of a multidisciplinary approach to medicine. A combination of her pediatrician, emergency physician, hospitalist, radiology, nephrology, and more was imperative to her receiving appropriate care. With the prophylactic antibiotics keeping infection at bay, watchful waiting is appropriate, and surgery can be considered if infections recur.

## Conclusions

UTIs in children can lead to life-threatening sepsis if left untreated. Prompt diagnosis and understanding of the underlying cause can lead to appropriate medical treatment and supportive care. When the patient has VUR, prophylaxis is needed due to how easily urine can flow in retrograde. Infection can easily climb to the kidneys leading to pyelonephritis and urosepsis. Each patient with VUR will require a personalized treatment plan to avoid infection while also avoiding unnecessary procedures. Recurrent fevers and infections should always be further evaluated, especially in children.
